# Systemic autoimmunity induced by the TLR7/8 agonist Resiquimod causes myocarditis and dilated cardiomyopathy in a new mouse model of autoimmune heart disease

**DOI:** 10.1242/dmm.027409

**Published:** 2017-03-01

**Authors:** Muneer G. Hasham, Nicoleta Baxan, Daniel J. Stuckey, Jane Branca, Bryant Perkins, Oliver Dent, Ted Duffy, Tolani S. Hameed, Sarah E. Stella, Mohammed Bellahcene, Michael D. Schneider, Sian E. Harding, Nadia Rosenthal, Susanne Sattler

**Affiliations:** 1 The Jackson Laboratory, 600 Main Street, Bar Harbor, ME 04609, USA; 2 Biological Imaging Centre, Department of Medicine, Imperial College London, London W12 0NN, UK; 3 Centre for Advanced Biomedical Imaging, Division of Medicine, University College London, London WC1E 6DD, UK; 4 National Heart and Lung Institute, Imperial CollegeLondon, London W12 0NN, UK

**Keywords:** Autoimmunity, Dilated cardiomyopathy, Heart disease, Myocarditis, Model, Resiquimod, Toll-like receptor 7/8

## Abstract

Systemic autoimmune diseases such as systemic lupus erythematosus (SLE) and rheumatoid arthritis (RA) show significant heart involvement and cardiovascular morbidity, which can be due to systemically increased levels of inflammation or direct autoreactivity targeting cardiac tissue. Despite high clinical relevance, cardiac damage secondary to systemic autoimmunity lacks inducible rodent models. Here, we characterise immune-mediated cardiac tissue damage in a new model of SLE induced by topical application of the Toll-like receptor 7/8 (TLR7/8) agonist Resiquimod. We observe a cardiac phenotype reminiscent of autoimmune-mediated dilated cardiomyopathy, and identify auto-antibodies as major contributors to cardiac tissue damage. Resiquimod-induced heart disease is a highly relevant mouse model for mechanistic and therapeutic studies aiming to protect the heart during autoimmunity.

## Abstract (precis)

**Summary:** A novel mouse model of autoimmune-mediated heart damage to study the underlying mechanisms and test therapeutic options for systemic autoimmunity.

## INTRODUCTION

The immune system is crucially involved in the maintenance of tissue homeostasis and the response to tissue damage. However, when it is activated by self-antigens, the immune system causes tissue damage itself and leads to autoimmune disease. Autoimmune and cardiovascular diseases are generally considered to affect distinct demographic groups. However, autoimmune patients have a substantially increased risk of developing cardiovascular complications, as well as a significantly worse prognosis after myocardial infarct compared with the general population (McCoy et al., 2013). Pharmacological treatment of autoimmune patients naturally focuses on the management of the autoimmune condition; however, the major cause of death in autoimmune patients is in fact related to cardiovascular problems (Jastrzębska et al., 2013). Involvement of the cardiovascular system in systemic autoimmune disease results from direct target-specific tissue damage due to autoreactive effector cells and antibodies, as well as from indirect damage due to increased systemic levels of inflammatory cytokines (Abou-Raya and Abou-Raya, 2006; Abusamieh and Ash, 2004; Knockaert, 2007).

The most common cardiac complication of the prototype systemic autoimmune disease systemic lupus erythematosus (SLE) is pericarditis, followed by myocarditis or myocardial fibrosis due to infiltration of inflammatory cells (Jastrzębska et al., 2013). Myocarditis may be idiopathic, infectious or autoimmune in origin. Inflammation may resolve or persist and lead to cardiac remodelling, ventricular dilation with normal or reduced left ventricular wall thickness, and systolic dysfunction, which in turn, leads to dilated cardiomyopathy (DCM) (Kawai and Matsumori, 2013; Magnani and Dec, 2006). Histology on DCM patient biopsies, shows myocyte loss, compensatory hypertrophy, fibrous tissue and chronic inflammation (myocarditis) in 30-40% of cases (Caforio et al., 2013). Over time, myocarditis may develop into dilated cardiomyopathy and heart failure (Baldeviano et al., 2010; Cihakova and Rose, 2008; Eriksson and Penninger, 2005; Knockaert, 2007), and is therefore a significant source of morbidity and mortality.

Epicutaneous application of the Toll-like receptor (TLR) 7/8 agonist Resiquimod (R848) has recently been presented as a new model for SLE (Yokogawa et al., 2014). The authors elegantly demonstrated the presence of auto-antibodies and multi-organ involvement. A role for plasmacytoid dendritic cells (pDC) and TLR7 was confirmed by *in vivo* pDC depletion studies and studies using TLR7-deficient mice, respectively. The authors suggested that interferon (IFN)-α production by skin-infiltrating plasmacytoid dendritic cells, which promotes B-cell expansion and maturation and activates myeloid cells and autoreactive T-cells, as the underlying mechanism for the induction of systemic autoreactivity (Yokogawa et al., 2014).

Here, we present a thorough functional, phenotypic and mechanistic characterisation of the cardiac effects of Resiquimod-induced autoimmunity to establish an inducible mouse model for cardiac involvement in systemic autoimmune disease and introduce a new mouse line with increased susceptibility compared with parental strains.

## RESULTS

### Resiquimod-induced systemic autoimmunity causes left ventricular dilation and affects heart function

A recombinant inbred mouse line derived from C57BL/6J, FVB/NJ and NOD/ShiLtJ parental lines was treated with Resiquimod to induce systemic autoimmunity. This mouse line, hereafter referred to as CFN, was obtained initially as a control for a different study and showed notable sensitivity to Resiquimod with clinical signs including anaemia (pale skin), skin haemorrhages, decreased urinary output, reduced mobility, piloerection, increased breathing rate and hunched posture after only 2 weeks of treatment. This was striking, as the authors of the first report of Resiquimod treatment to induce systemic autoimmunity (Yokogawa et al., 2014) described treatment durations of up to 8 weeks in FVB/NJ and BALB/C mouse strains. Importantly, these signs of acute disease were transient and resolved within a few days. We therefore used three Resiquimod applications per week for 2 weeks as standard treatment in all subsequent experiments.

**Fig. 1.**
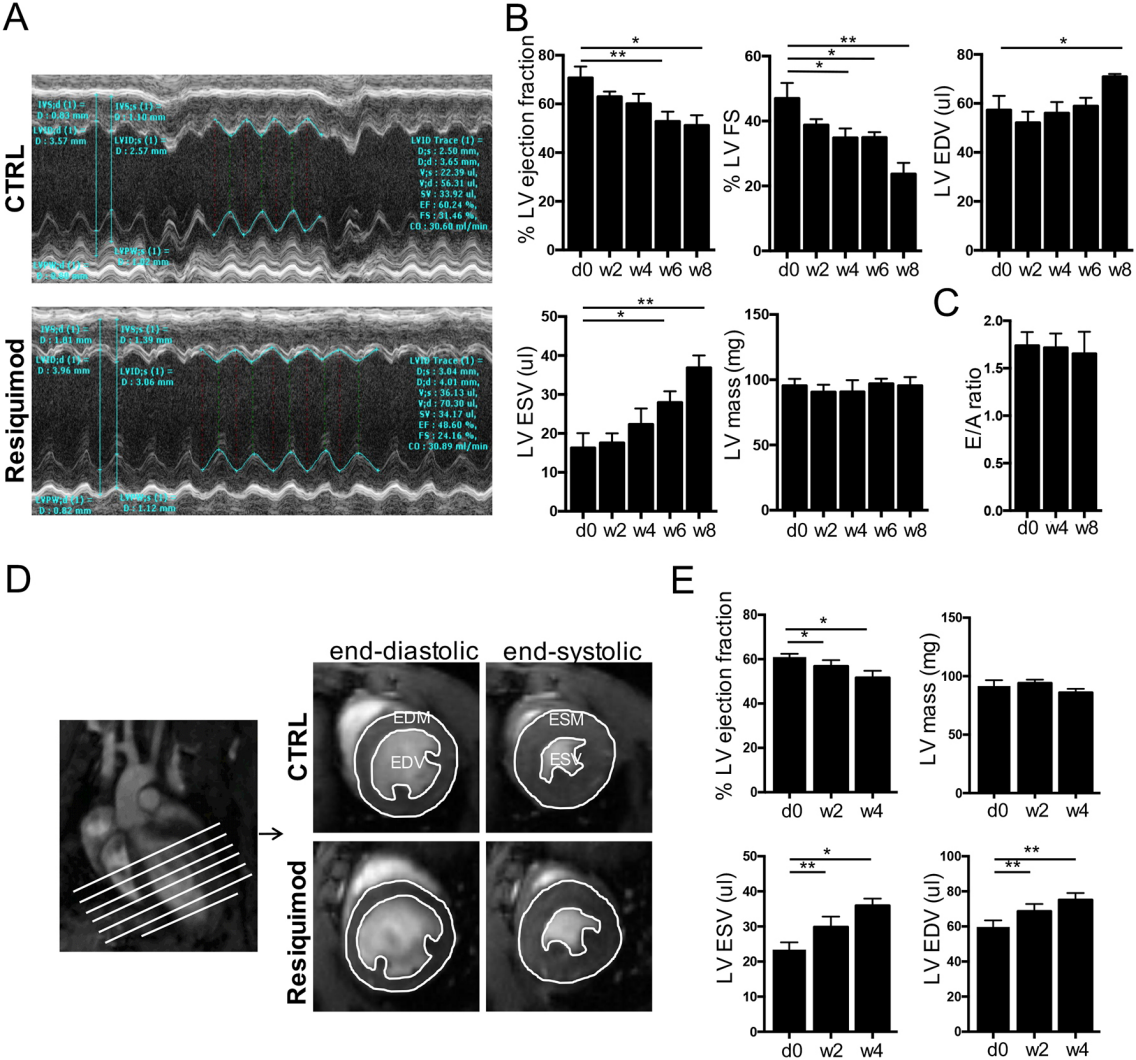
**Resiquimod treatment induces morphological changes and functional impairment of the heart.** (A) Examples of m-mode traces of a heart from a Resiquimod-treated mouse compared with traces of a control mouse heart. (B) Quantification of LV end-systolic and end-diastolic volumes (ESV and EDV), LV mass, fractional shortening and ejection fraction in Resiquimod-treated mice measured at baseline, and at 2 week intervals until week 8. (C) Diastolic function of Resiquimod-treated mice over time assessed by Doppler echocardiography measurements of early (A) and late (E) ventricular filling velocities across the mitral valve. For B and C *n*=8/time point (week 8: *n*=3), data pooled from two independent repeats. (D) Cardiac MR images demonstrating how parameters of cardiac structure and function were assessed. 10-frame movies including end diastolic and end systolic images of 7 cross-sections (white lines) from base to apex were acquired and segmented to obtain the outer and inner border of the LV to determine end diastolic and end systolic mass (EDM, ESM), and EDV and ESV, as well as ejection fraction. (E) Quantification of LV ESV and EDV, LV mass and ejection fraction in Resiquimod-treated mice measured at baseline and 2 and 4 weeks after the start of treatment. *n*=5/group, two independent repeats. Values represent mean±s.e.m.; *P<0.05, **P<0.005, one-tailed, paired Student's *t*-test.

To determine if the Resiquimod model was suitable to investigate cardiac involvement in systemic autoimmune disease, cardiac function upon treatment was measured by echocardiography and magnetic resonance imaging (MRI; Fig. 1). CFN mice were treated for 2 weeks with Resiquimod and monitored by echocardiography over 2 week intervals for up to 8 weeks (Fig. 1A,B). They showed a consistent decrease in left ventricular (LV) ejection fraction and fractional shortening (FS), as well as an increase in LV end-systolic and end-diastolic volumes (ESV and EDV, respectively), while LV mass remained largely unchanged (Fig. 1B). Diastolic function assessed by Doppler echocardiography measurements of early (A) and late (E) ventricular filling velocities across the mitral valve also remained unchanged (Fig. 1C) and no signs of conduction abnormalities were observed on electrocardiograms (Fig. S1).

As echocardiography yielded significant differences only at later stages, we performed MRI, which is considered a more accurate method for assessment of cardiac function (Stuckey et al., 2008), to detect subtle differences at earlier stages (Fig. 1D,E). A significant drop in ejection fraction was detectable as early as week 2 after the start of treatment. In line with echocardiography results, we observed a striking increase in LV volume, whereas LV mass remained unchanged (Fig. 1E). The morphological changes in the LV culminate in functional impairment, as shown by progressively decreased fractional shortening (measure of LV stiffness and contractility) and a reduced ejection fraction (measure of LV contractility).

These features of myocardial dysfunction and LV dilation with largely unchanged LV mass are indicative of dilated cardiomyopathy, and have been observed in patients with underlying autoimmune disease. Dilated cardiomyopathy can develop secondary to myocarditis (Cihakova and Rose, 2008; Eriksson and Penninger, 2005), and has been observed in SLE, systemic sclerosis, dermatomyositis and Churg–Strauss syndrome (Chen et al., 2014; Elliott, 2000; Jeong et al., 2015; Quartier et al., 2002; Zhang et al., 2012).

### Resiquimod-mediated inflammation induces severe cardiac tissue damage and recapitulates cardiac symptoms of human systemic autoimmune disease

**Fig. 2.**
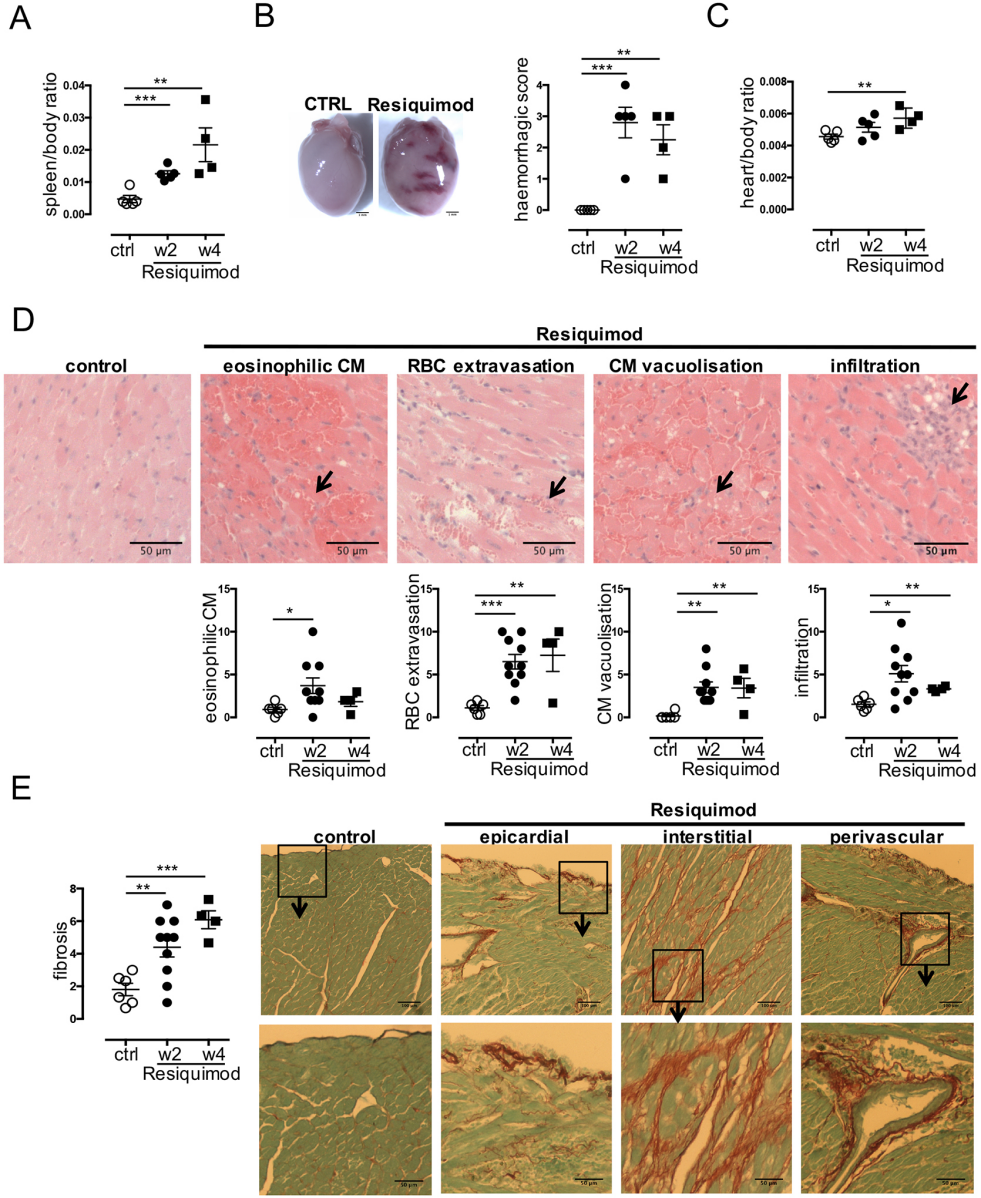
**Resiquimod treatment induces cardiac tissue damage as seen in autoimmune disease.** (A) Splenomegaly as a measure of systemic immune activation 2 and 4 weeks after start of treatment. (B) Macroscopic observation of morphological changes, severe hyperaemia and haemorrhagic lesions of hearts *ex vivo* 2 weeks and corresponding quantification 2 and 4 weeks after start of treatment. Macroscopic haemorrhagic lesions were scored on a scale according to: no lesions (=); lesions cover <10% of heart surface (1); lesions cover 10-30% of heart surface (2); lesions cover 30-50% of heart surface (3); lesions cover >50% of heart surface (4). (C) Heart/body mass ratio 2 and 4 weeks after start of treatment. (D) H&E-stained paraffin-embedded heart sections (top) showing eosinophilic cardiomyocytes, islands of hyperaemia and extravasation of red blood cells (RBC), cardiomyocyte (CM) vacuolisation and mononuclear cell infiltration as indicated by black arrows 400× magnification. Original micrograph is cropped to show an example of the respective damage parameter. Corresponding semi-quantitative scores are shown below. (E) PicoSirius Red-stained frozen heart sections showing epicardial, interstitial and perivascular fibrosis. Top row: 200× magnification; bottom row, 3.3× magnification of area of interest in panel above. Individual cardiac damage parameters were scored on a scale from 0 to 3 (none, mild, moderate, severe) in five fields of view in four heart cross-sections at papillary muscle level per mouse. *n*=4-10/group (each symbol represents one individual mouse), one representative experiment shown of >3 independent repeats. Values represent mean±s.e.m. *P<0.05, **P<0.005 and ***P<0.001, two-tailed, unpaired Student's *t*-test.

Yokogawa et al. (2014) suggested that Resiquimod treatment induces cardiac tissue damage as part of the systemic autoimmune syndrome. As cardiac damage and the resulting phenotype were not studied in detail, we aimed to characterise its molecular and cellular aetiology. Upon necropsy, mice treated with Resiquimod showed severe splenomegaly (Fig. 2A) and enlarged hearts with a rounded shape and remarkable haemorrhagic lesions as early as 2 weeks after treatment (Fig. 2B). Initial *in vivo* imaging results (Fig. 1) showing dilation of the left ventricle rather than thickening of the cardiac muscle (hypertrophy), are congruent with a comparably minor increase in heart/body mass ratio at later stages, compared with untreated mice (Fig. 2C). Histopathological analysis (Fig. S2) of the hearts revealed significant damage to the whole heart, most prominently in the endocardium and myocardium and the papillary muscles. Cardiomyocyte damage and cell death was evident through (1) increased intensity of eosinophilic staining and fragmentation (cardiomyocyte apoptotic bodies) (Beranek, 2001), (2) cardiomyocytes with intracellular vacuolisation, (3) oedema and (4) red blood cell extravasation, indicating capillary damage and immune cell infiltration (Fig. 2D), as well as (5) epicardial, interstitial and perivascular fibrosis (Fig. 2E). These features resemble autoimmune pancarditis in SLE patients, which is characterised by interstitial oedema, immune cell infiltration into the myocardial interstitium, epicardium and endocardium, and areas of myocyte necrosis and fibrosis (Bulkley and Roberts, 1975; Busteed et al., 2004; Duan et al., 2013; Salomone et al., 1989). Acute haemorrhagic myocarditis has also been reported as a complication in SLE (Dickens et al., 1992). Myocardial lesions with necrosis and replacement fibrosis may progress to chronic myocarditis and dilated cardiomyopathy (Wijetunga and Rockson, 2002).

The Resiquimod model is thus a suitable model to study all stages of heart disease secondary to systemic autoimmunity, starting from acute myocarditis to later chronic stages of cardiac remodelling towards LV dilation and heart failure.

### Resiquimod treatment induces a cellular immune response dominated by lymphocytes in the heart and peripheral lymphoid organs

**Fig. 3.**
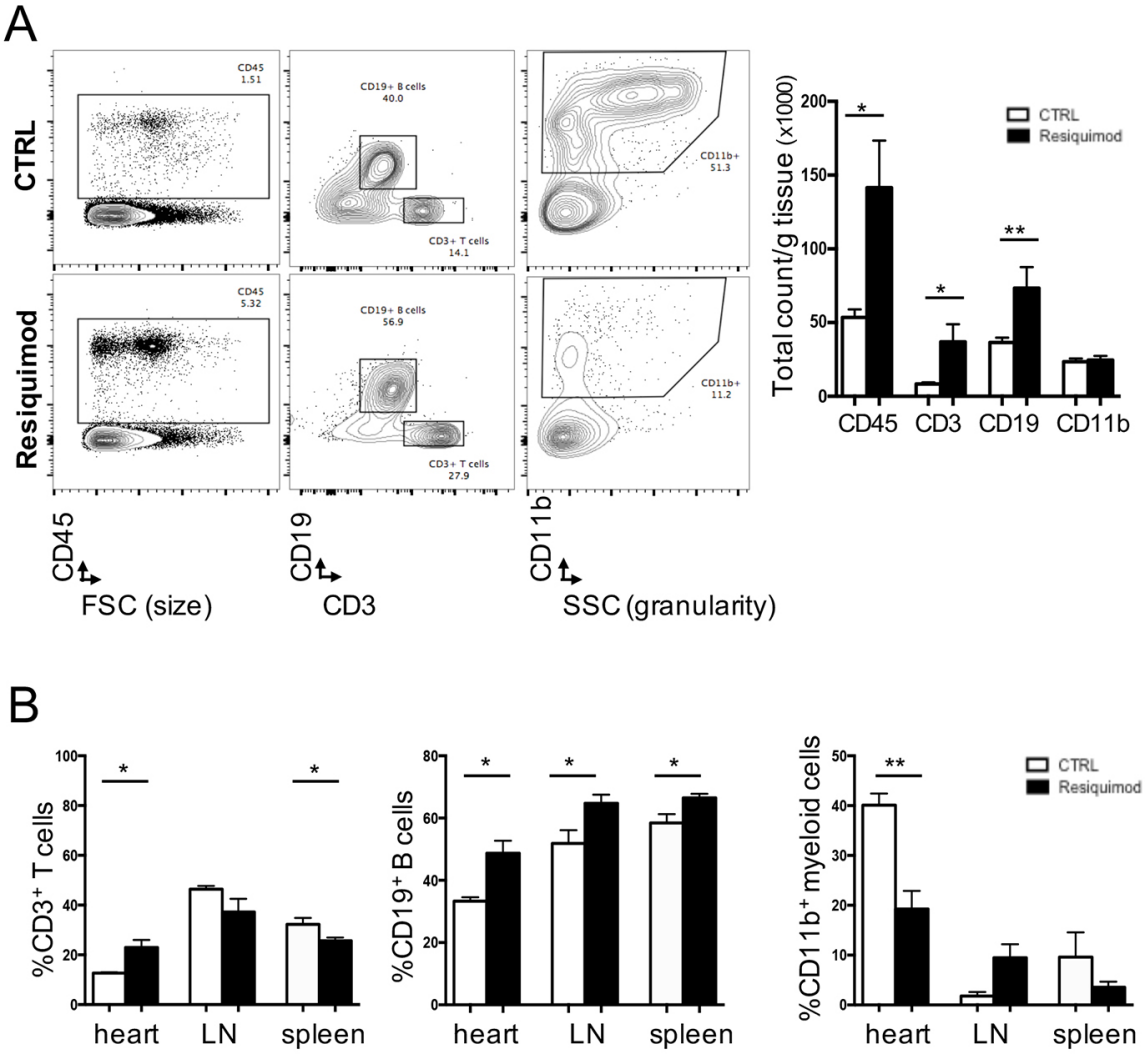
**The Resiquimod-induced cellular immune response in the heart, mediastinal lymph nodes and spleen.** (A) Representative blots obtained by flow cytometry showing differences in immune cell populations in the hearts of control and Resiquimod-treated mice (left) and corresponding total counts of CD45^+^, CD3^+^, CD19^+^, CD11b^+^ immune cells/g heart tissue in Resiquimod-treated versus untreated mice (right). (B) Frequency of CD3^+^, CD19^+^, CD11b^+^ immune cells among total CD45^+^ immune cells in heart tissue, mediastinal lymph nodes and spleens in Resiquimod-treated versus untreated mice. *n*=4/group, data pooled from two independent experiments. Values represent mean±s.e.m. *P<0.05 and **P<0.005, two-tailed, unpaired Student's *t*-test.

One of the salient effects of an autoimmune response is an increase of immune cell infiltration into affected organs and tissues (Askanas et al., 1994; Dosreis, 1999; Koo et al., 2013; Lafyatis et al., 2007; Sun et al., 2013). To determine the extent to which Resiquimod treatment causes immune cells to infiltrate the heart, which consequently could be directly responsible for the observed cardiac damage, CFN mice were treated with Resiquimod for 2 weeks and the heart, mediastinal lymph nodes and spleens were harvested at 1 week post-treatment for flow cytometric analysis. In the heart, the total number of CD45^+^ haematopoietic cells per gram of tissue increased upon treatment with Resiquimod. This was largely due to increased CD19^+^ B-cell and CD3^+^ T-cell numbers, while overall levels of CD11b^+^ myeloid cells were unaffected (Fig. 3A). Further analysis of the heart and peripheral immune organs showed a relative increase in the proportion of lymphocytes and a significantly reduced proportion of myeloid cells among hematopoietic cells (Fig. 3B), indicating that the adaptive rather than the innate immune system is involved in the Resiquimod-mediated direct cardiac damage. One possible cellular mechanism to explain this observation is an activation of lymphoid cells in the peripheral immune organs, followed by migration into the heart.

Although differences did not reach statistically significant levels, an overall increase in relative numbers of CD11b^+^ cells in the mediastinal lymph nodes was observed, suggesting that myeloid cells are involved in activation of B-cells and T-cells in the lymph node, which then migrate and accumulate in the heart. While application of Resiquimod has been shown to reduce marginal zone B-cells in the spleen and to increase the activated B-cell population (Yokogawa et al. 2014), the data presented here suggest the involvement of the adaptive immune system in inducing cardiac damage.

### Resiquimod-mediated systemic autoimmunity increases levels of anti-cardiac antibodies in the serum, which are deposited in the heart

**Fig. 4.**
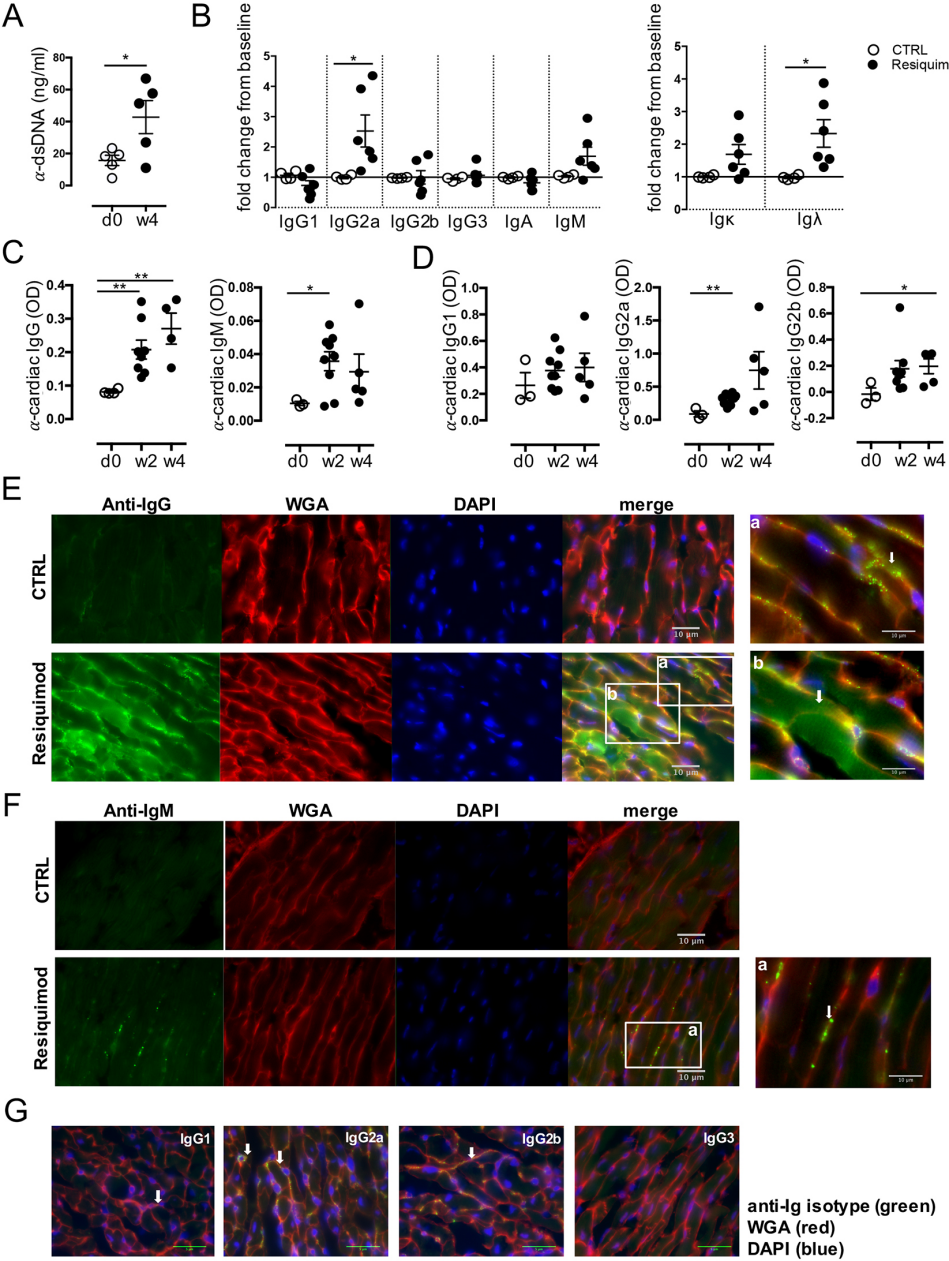
**Resiquimod induces anti-cardiac auto-antibodies.** (A) Levels of anti-dsDNA at week 4 after Resiquimod treatment. (B) ELISA to detect relative levels of antibody isotypes as indicated (light and heavy chains) in serum of untreated and Resiquimod-treated mice. Data pooled from two independent experiments. (C) Levels of anti-cardiac auto-antibodies of IgM and IgG isotype. (D) Levels of anti-cardiac auto-antibodies with IgG1, IgG2a and IgG2b heavy chains. (E-G) Immunofluorescence staining using anti-mouse IgG-Alexa Fluor 488 (E), anti-mouse IgM-FITC (F), anti-mouse IgG1/IgG2a/IgG2b/IgG3 and a secondary anti-rat IgG-Alexa Fluor 488 (G) to detect *in vivo* deposited antibodies and immuno-complexes as indicated by white arrows. Green: IgG/IgM/IgG1/IgG2a/IgG2b/IgG3 staining; red: wheat germ agglutinin staining membranes; blue: DAPI staining of nuclei. 400× original magnification. Areas of interest (a,b in E and a in F): additional 2× magnification. *P<0.05, **P<0.005, two-tailed, paired/unpaired Student's *t*-test.

While increased infiltration may not necessarily reflect an active pathological immune response against the affected organ, the presence of pathogenic anti-self antibodies is a hallmark of autoimmune disease (Suurmond and Diamond, 2015). The Resiquimod-induced SLE model has been demonstrated to develop auto-antibodies (Yokogawa et al., 2014) and anti-dsDNA auto-antibodies can be detected in the serum of Resiquimod-treated CFN mice (Fig. 4A). To define the potential pathogenic role of autoreactive B-cells and auto-antibodies in cardiac damage, serum from Resiquimod-treated mice was analysed for cardiac autoreactivity and antibody isotype composition. Different antibody isotypes elicit different immune effector functions and the presence of specific isotypes such as IgG2a and IgG2b has been correlated with pathological immune responses in autoimmune disease (Ehlers et al., 2006; Lleo et al., 2010). We therefore analysed the change in isotype composition of the circulating antibody repertoire in Resiquimod-treated animals before and after treatment by using an ELISA to detect IgG1, IgG2a, IgG2b, IgG3, IgA and IgM as well as the light chains Igκ and Igλ. While the antibody isotype repertoire did not change in untreated animals, an increase in IgM and IgG2a was evident in Resiquimod-treated mice. In addition, in line with an increase in total antibody levels, both Igκ and Igλ light chain levels increased dramatically (Fig. 4B; Fig. S3). Most importantly, levels of auto-antibodies of both IgM and IgG isotypes that were reactive to cardiac lysate quickly increased in the serum after the start of treatment and were significantly elevated over background levels by 2 weeks (Fig. 4C). In particular, anti-cardiac IgG2a, and later, IgG2b antibodies, were significantly elevated in treated CFN mice (Fig. 4D). Cardiac myosin and troponin were identified as two specific antigens targeted by auto-antibodies in serum of Resiquimod-treated mice (Fig. S5).

To confirm that anti-cardiac antibodies have the ability to reach and bind cardiac structures *in vivo*, we performed immunofluorescence staining of IgG isotypes and IgM in frozen heart sections of Resiquimod-treated mice. We found abundant *in vivo* deposition of IgG immune complexes, as shown by a focal punctate staining along the cardiomyocyte sarcolemma (Fig. 4E, panel a) as well as overall hazy staining in potentially damaged cardiomyocytes with a stronger staining along the sarcomeres (Fig. 4E, panel b). Less abundant IgM complexes followed the same focal pattern along the sarcolemma as IgG complexes (Fig. 4F). Interestingly, there was no detectable complement C3c deposition in the heart, although both IgG and C3c were readily detectable in the kidneys (Fig. S4), indicating that C3-independent mechanisms of damage might be involved. Corresponding to the expansion of IgG2a and IgG2b isotype antibodies in the serum, the prominent IgG subtypes deposited in damaged cardiac tissue, contained IgG2a and IgG2b heavy chains. Very mild staining for IgG1 with a cellular appearance rather than bright antibody complexes was detectable, while no IgG3 was found (Fig. 4G). In summary, the observed increase in B-cell proliferation, with accompanying accumulation of post-class switching antibodies that bind to cardiac structures *in vivo*, suggests a pathological role for the auto-antibody pool.

### Resiquimod-mediated cardiac damage is inducible by adoptive transfer of splenocytes and serum

**Fig. 5.**
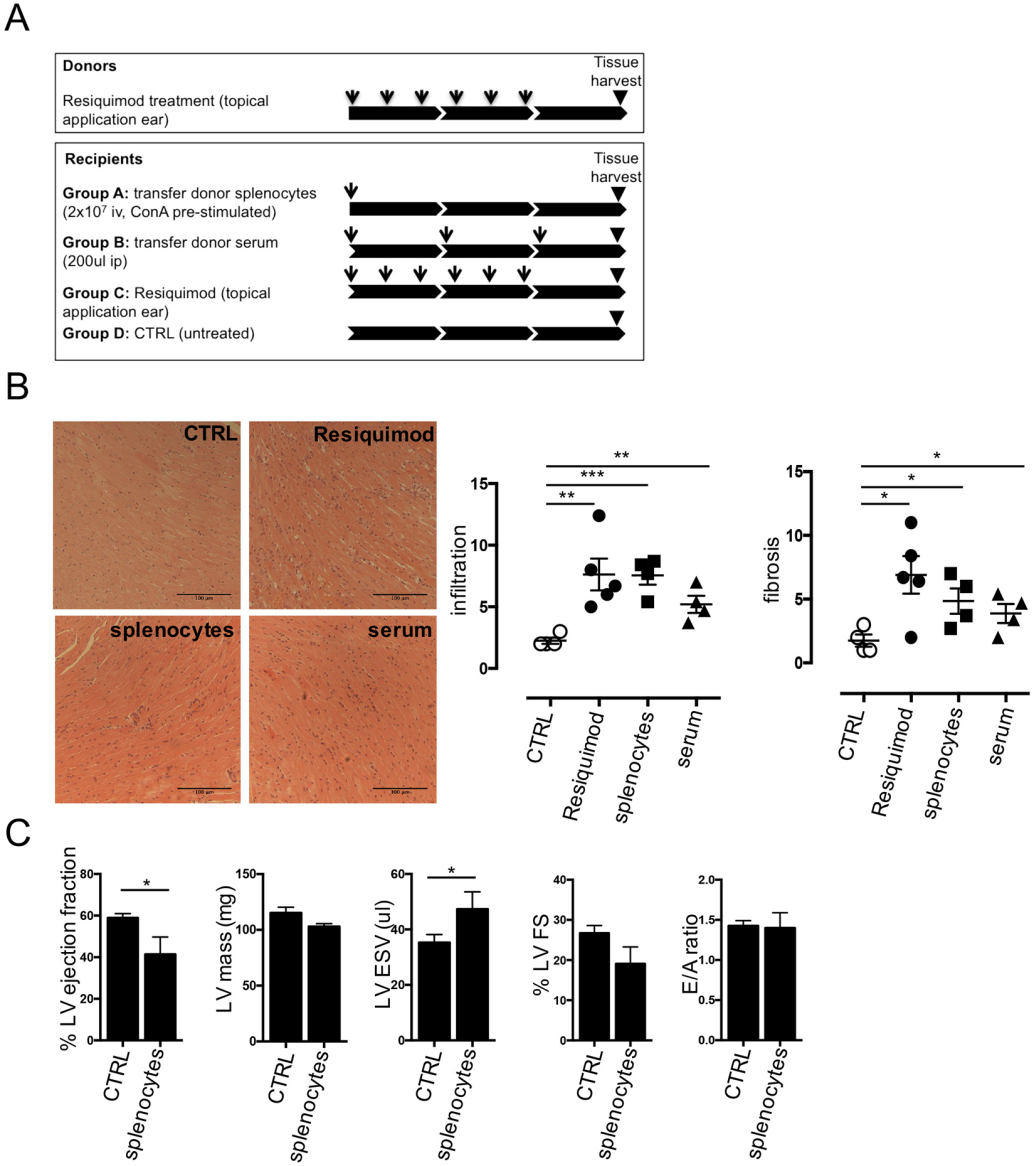
**Transfer and mutant studies indicate that adaptive immune cells induce cardiac damage.** (A) Donor mice were treated with Resiquimod as described above and spleens and sera were harvested one week after cessation of treatment. 2×10^7^ splenocytes/recipient mouse were pre-stimulated *in vitro* with 5 μg/ml concanavalin A for 2 days and injected via the tail vein. 200 μl serum/mouse was injected three times per mouse 1 week apart. Control mice were either treated with Resiquimod as per standard 2 week treatment protocol or left untreated. Hearts of recipient and control mice were harvested 3 weeks after start of treatment. (B) H&E- or PicoSirius Red-stained paraffin-embedded heart sections (representative examples) were scored for degree of mononuclear cell infiltration and fibrosis on a scale from 0-3 (none, mild, moderate, severe) in five fields of view in four heart cross-sections at papillary muscle levels per mouse. (C) Systolic and diastolic function of ‘Group A’ splenocyte recipient mice 4 weeks after splenocyte transfer. Quantification of LV end-systolic and end-diastolic volumes, LV mass, fractional shortening and ejection fraction measured at baseline, and after 4 weeks. *n*=3/time point. Diastolic function assessed by Doppler echocardiography measurements of early (A) and late (E) ventricular filling velocities. *n*=3/time point. Values represent mean±s.e.m. *P<0.05, **P<0.005 and ***P<0.001, two-tailed, paired/unpaired Student's *t*-test.

The increase of IgG2a and IgG2b molecules in the serum and immune complex deposition in the hearts of Resiquimod-treated animals strongly indicate a central role of B-cells, as seen in many systemic autoimmune conditions (Browning, 2006; Lleo et al., 2010). To distinguish between cellular and humoral effectors in this response, we treated donor CFN mice for 2 weeks, and harvested the spleens and sera 1 week post-treatment. Donor splenocytes were pre-stimulated *in vitro* with Concanavalin A following a protocol for T-cell-dependent transfer of myosin-induced myocarditis (Myers et al., 2013) and injected intravenously into recipient mice. Donor sera were injected into a separate group of recipient mice following a slightly modified protocol of passive serum transfer (Bonney et al., 2013). As controls, donor-cell-free mice were either left untreated (negative control) or treated with Resiquimod (positive control). Fig. 5A illustrates the experimental design.

Strikingly, splenocyte transfer faithfully recapitulated the Resiquimod-induced heart disease phenotype. Injection of antibody-containing serum from Resiquimod mice produced the same qualitative pattern of damage, albeit with a milder response (Fig. 5B). Splenocyte transfer also recapitulates morphological and functional changes in the heart, with significant LV dilation and a reduction in ejection fraction 4 weeks after the start of treatment (Fig. 5C). Notably, effects on heart morphology and function seemed to be more pronounced and homogenous between treated individuals compared with direct Resiquimod treatment. This not only proves the potent involvement of cellular immunity in the induction of heart disease, but also provides an alternative method to induce Resiquimod-mediated damage, as well as the basis for a thorough investigation of the cell populations involved.

Since cells and sera were harvested from donor mice 1 week after Resiquimod treatment, transfer of the agent from the donor to the recipient was unlikely. While it cannot be excluded that other serum factors are involved in disease induction, specific anti-cardiac antibodies (Fig. 4) are a very likely aetiology of disease transmission.

### Genetic differences determine immune responses to Resiquimod treatment and the severity of cardiac damage

**Fig. 6.**
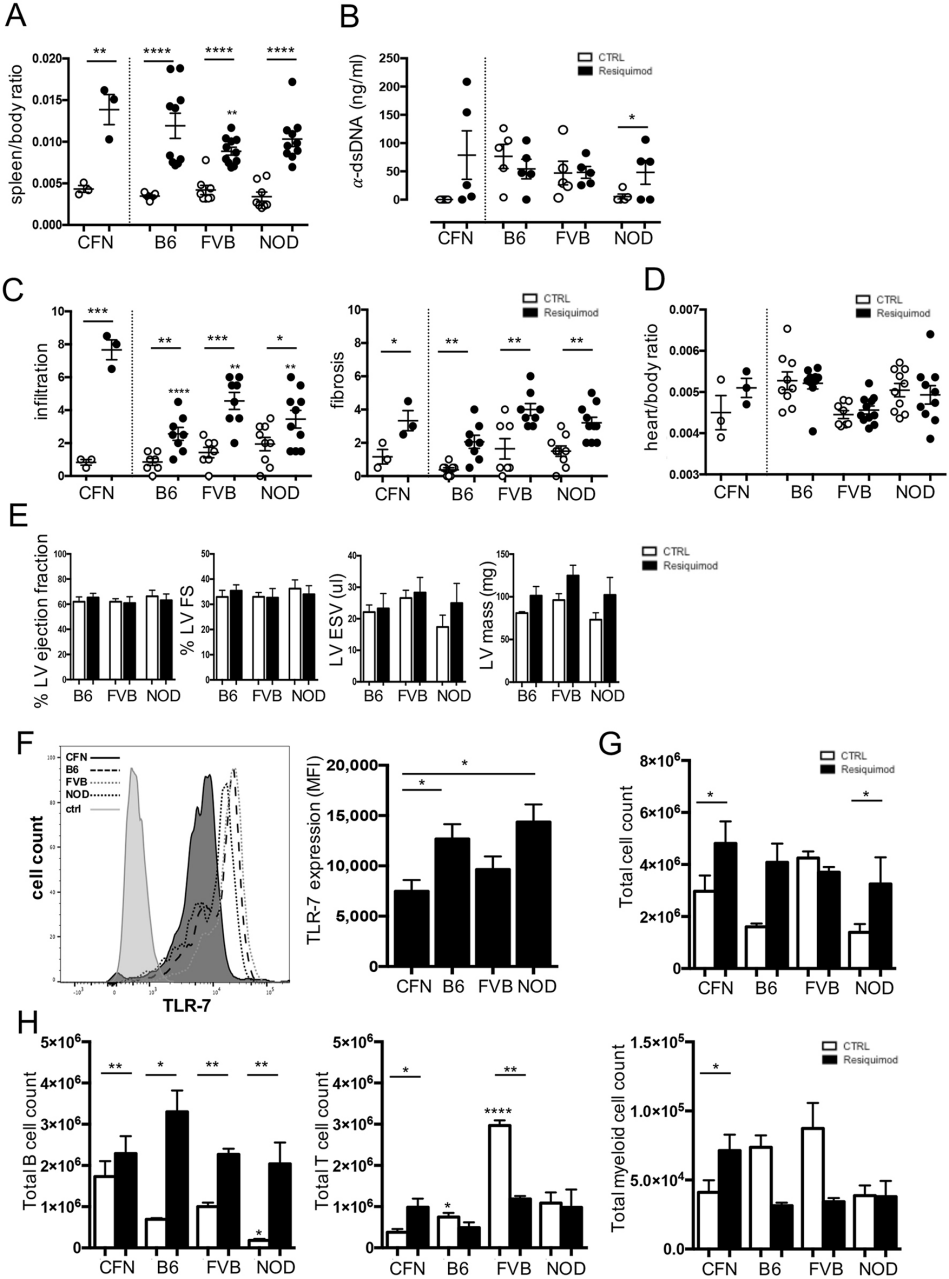
**Genetic variation defines the responses to Resiquimod treatment and the severity of cardiac damage.** (A) Splenomegaly as a measure of systemic immune activation 2 weeks after start of treatment in CFN, C57Bl/6J (B6), FVB/NJ (FVB) and NOD/ShiLtJ (NOD). (B) Levels of anti-dsDNA at week 2 after Resiquimod treatment. (C) Semi-quantitative scores of degree of mononuclear cell infiltration and fibrosis based on H&E- and PicoSirius Red-stained paraffin-embedded heart sections. Individual damage parameters were scored on a scale from 0-3 (none, mild, moderate, severe) in five fields of view in four heart cross-sections in the myocardium at papillary muscle level per mouse. (D) Heart/body mass ratio 2 weeks after start of treatment. (E) Quantification of LV end-systolic volumes, LV mass, fractional shortening and ejection fraction in Resiquimod-treated mice measured at baseline (CTRL) and 6 weeks after start of treatment (Resiquimod). *n*=5. (F) Expression level of TLR7 in splenocytes as measured by mean fluorescence intensity (MFI) using flow cytometry; *n*=5. (G) Total cell count in culture at day 4 of splenocytes stimulated with 1 mg/ml Resiquimod. (H) Total T-cell, B-cell and myeloid cell counts in culture at day 4 in splenocytes stimulated with 1 mg/ml Resiquimod obtained by manual counting and flow cytometry staining against CD45^+^, CD3^+^, CD19^+^, CD11b^+^ immune cells; *n*=3. Values represent mean±s.e.m. *P<0.05, **P<0.005, ***P<0.001 and ****P<0.0001, two-tailed, unpaired Student's *t*-test. Asterisks directly above individual columns indicate a significant difference between the respective parental strain and CFN mice.

Cardiac damage was observed first in mixed-background CFN mice. To investigate which parental strain might have been the source of high susceptibility to cardiac damage, parental strains of C57Bl/6J, FVB/NJ and NOD/ShiLtJ genetic background were also treated using the 2 week Resiquimod regime optimised for CFN mice. All three parental strains responded with severe systemic inflammation as measured by a substantial increase in spleen/body mass ratio (Fig. 6A). However, increased levels of anti-dsDNA antibodies over baseline are only detectable in CFN and NOD/ShiLtJ strains 2 weeks after the start of treatment (Fig. 6B). Most importantly, although the degree of cardiac damage varied and did not correlate with the degree of systemic disease severity, all strains did develop significant inflammatory infiltration and fibrosis in their hearts (Fig. 6C). For example, while C57Bl/6J showed the highest increase in spleen/body mass ratio, immune cell infiltration into the heart was lower as compared with the other strains. Comparable to CFN mice, none of the parental strains had a detectable increase in heart/body mass ratio by week two (Fig. 6D), although the hearts appeared enlarged at necropsy. Notably, none of the parental strains developed significant functional impairments. Mild morphological changes indicative of the onset of LV dilation were observed in individual mice, particularly of the NOD/ShiLtJ background, but these did not reach statistical significance (Fig. 6E). These data indicate that there are differences in both the sensitivity to induction of systemic autoimmunity as well as susceptibility to a subsequent effect on the heart. There is no direct linear correlation between the degree of initial systemic inflammation, the ensuing cardiac tissue damage and the subsequent functional defects when comparing mice of different genetic backgrounds.

Considering the importance of variations in TLR7 levels for the susceptibility to autoimmune disease in humans, a potential involvement of TLR7 in the differences in susceptibility to Resiquimod-induced heart disease between strains was investigated. There are currently no single nucleotide polymorphisms known between the parental strains that could be predicted to cause changes in protein expression. Strikingly, flow cytometry showed that CFN mice expressed lower levels of TLR7 in splenocytes compared with the parental strains C57Bl/6J and NOD/ShiLtJ (Fig. 6F). A highly variable response of splenocytes to Resiquimod stimulation was observed *in vitro* between all four strains (Fig. 6G). However, all three parental strains showed a strong proliferative response of B-cells while numbers of T-cells and myeloid cell populations were maintained at baseline levels or decrease. In contrast, and in line with lower levels of TLR7 expression, CFN mice showed a less-pronounced increase in B-cell numbers, but also expanded numbers of T-cells and myeloid cells in the same culture (Fig. 6H).

Thus, in contrast to our expectations, increased levels of TLR7 expression and generalised immune hyper-responsiveness could not be detected. Instead, the unique response of CFN splenocytes to *in vitro* Resiquimod stimulation, comprising an overall lower expansion in cell numbers, but a parallel increase in B-cells, T-cells and myeloid cells, may provide a mechanistic basis for the observed differences on morphological and functional levels.

## DISCUSSION

Several models of primary heart-specific autoimmunity are well established and widely used, such as experimental autoimmune myocarditis (EAM) induced by immunisation with cardiac myosin, and Coxsackie virus B3-induced myocarditis (Ciháková et al., 2004). However, the field lacks a clinically relevant inducible model of heart disease that develops secondarily to systemic autoimmunity. Two models used to induce systemic autoimmunity, namely injection of pristane (Reeves et al., 2009) and chronic graft versus host disease (cGVHD) (Tyndall and Dazzi, 2008) have not been investigated for cardiac damage, and are compromised by their long and variable period until onset of symptoms (pristane), and severe restrictions due to genetic background, respectively. (cGVHD).

Resiquimod-induced systemic autoimmunity caused a morphological and functional cardiac phenotype that resembled immune-mediated myocarditis and dilated cardiomyopathy in a variety of autoimmune conditions, including SLE, systemic sclerosis, dermatomyositis and Churg–Strauss syndrome (Chen et al., 2014; Elliott, 2000; Jeong et al., 2015; Quartier et al., 2002; Zhang et al., 2012).

Histopathological analysis of the heart revealed oedema and red blood cell extravasation into the tissue, cardiomyocyte eosinophilia, vacuolisation and fragmentation, myofibre separation, immune cell infiltration and fibrosis. Oedema and interstitial red blood cells are a tell-tale sign of vessel wall damage, which allows passive leakage of fluids and cells into the tissue. This is probably an early phenomenon in the course of Resiquimod-induced cardiac damage, caused by the pro-inflammatory effects of systemically increased cytokine levels on endothelial and smooth muscle cells. Signs of damage to larger vessels in the myocardium were not observed; thus, damaged capillaries are the likely source of interstitial fluids and red blood cells. Oedema and red blood cell extravasation have also been observed in haemorrhagic myocarditis in patients with acute flares of systemic autoimmunity, thus making the model relevant for the human condition (Dickens et al., 1992). Cardiomyocyte necrosis, scattered interstitial immune cell infiltration and focal areas of severe damage with patches of immune cell accumulation and subsequent fibrosis, as seen in human autoimmune myocarditis and dilated hypertrophy, are also evident upon histological analysis (Bulkley and Roberts, 1975; Busteed et al., 2004; Duan et al., 2013; Salomone et al., 1989; Wijetunga and Rockson, 2002).

Immune cell infiltration into the myocardium may occur in response to damage in an attempt to remove debris and repair tissue, as seen after a myocardial infarct, or as a sign of active immune autoreactivity against previously healthy tissue structures as seen in autoimmune responses. Importantly, an immune response to tissue trauma follows a tightly controlled course of events, with an almost instant dramatic increase in innate immune cell numbers followed later by a reduced number of adaptive lymphocytes. It is resolved quickly in order to avoid additional inflammatory damage (Gallego-Colon et al., 2015; Ramos et al., 2016; Sattler and Rosenthal, 2016; Yan et al., 2013). By contrast, immune autoreactivity initiates a slow-onset vicious cycle of initially milder but widespread damage and repair attempts, perpetuating and exacerbating damage, inflammation and autoimmunity (Mackay et al., 2008). Thus, while the composition of the immune infiltrate after physical damage is generally defined by the exact time point after damage, the infiltrate resulting from an autoimmune response is characterised by the simultaneous presence of both active aggressors and passive responders, most likely at variable relative levels.

At 3 weeks post-Resiquimod treatment, which is presumably the peak of infiltration of adaptive immune aggressors, we observed a modest increase in total immune cell numbers in the heart, with a clear lymphocyte dominance, especially B-cells, in both total and relative numbers. Importantly, both B- and T-cells are important pathogenic components of several autoimmune diseases, including SLE, type-1 diabetes and rheumatoid arthritis (Askanas et al., 1994; Dosreis, 1999; Koo et al., 2013; Lafyatis et al., 2007; Sun et al., 2013). However, the role of B-cells in autoimmune disorders varies: in type-1 diabetes, they are predominantly professional antigen-presenting cells (Dörner et al., 2011; Serreze and Silveira, 2003; Wong et al., 2004), whereas in SLE and rheumatoid arthritis, B-cells contribute to pathology by auto-antibody production (Dörner et al., 2011; Marston et al., 2010). Thus the exact role of B-cells in cardiac damage still needs to be fully determined. Here, we show that mature, class-switched auto-antibodies against cardiac antigens, including two well-known targets for cardiac auto-antibodies – myosin and cardiac troponin I – are present in the serum of Resiquimod-treated mice. In addition, antibodies with mainly IgG2a and IgG2b heavy chains, which have been implicated in pathological immune responses in autoimmune disease (Ehlers et al., 2006; Lleo et al., 2010), are deposited in damaged hearts. Importantly, auto-antibodies against antigens in the cardiomyocyte contractile apparatus, such as troponin and myosin, may also cause cellular damage and induce a remodelling response in the myocardium by directly interfering with cardiomyocyte function (Kaya et al., 2012).

While anti-cardiac antibodies are prominent players, the additional contribution of antigen presentation or cytokine production by B-cells is likely. The *in vivo* transfer studies presented here show that both splenocytes and serum from Resiquimod-treated mice can directly induce damage, immune cell infiltration and fibrosis, and splenocytes also recapitulate functional effects on the heart. This confirms that the cellular and/or humoral arm of the immune system are indeed causative in induction of disease rather than secondary epiphenomena.

The role of myeloid cells in Resiquimod-mediated autoimmunity is less straightforward. In healthy myocardium, myeloid cells represent a large part of CD45^+^ cells (Pinto et al., 2016), and macrophages in particular have gained prominence as mediators of repair and regeneration after tissue damage (Pinto et al., 2014; Tonkin et al., 2015). While the total number of cardiac CD11b^+^ myeloid cells in the heart does not change in response to Resiquimod, a slight increase was observed in the mediastinal lymph nodes. It is therefore likely that Resiquimod treatment induces both lymphoid and myeloid anti-cardiac autoreactivity in the mediastinal lymph nodes, at which point predominantly autoreactive lymphoid cells migrate into the heart and cause tissue destruction. This scenario is reminiscent of the progression of type-1 diabetes, where the hub of the immune response is the pancreatic lymph node and the destruction occurs in the pancreas (Gagnerault et al., 2002). In the case of cardiac autoimmunity, local lymphocyte autoreactivity ensures an ongoing cycle of damage and fibrotic repair, leading to left ventricle adverse remodelling, dilation and a decline in heart function.

Another aspect making the presented model highly relevant to human autoimmune disease, is the impact of genetic variation on disease severity as observed when comparing mouse strains of different genetic backgrounds. Genetic variation not only defines the initial severity of systemic inflammation, but also determines the degree of secondary damage to the heart. The mixed-background CFN mouse strain has inherited a genome that is highly susceptible to immune-mediated cardiac damage and will therefore, together with its parental strains, be useful in investigating the underlying genetic basis of cardiac involvement in systemic autoimmunity.

In summary, investigation of the cellular and molecular components of the Resiquimod-induced cardiac autoimmunity shows that the cellular lymphoid compartment of the immune system and/or pathogenic auto-antibodies in the serum are primarily responsible for the observed phenotype in the heart. This mouse model is useful to study the cellular immunology of heart damage in systemic autoimmune disease and represents a tool to test novel therapies for immune-mediated myocarditis and dilated cardiomyopathy.

## MATERIALS AND METHODS

### Mice

Mice used were 8- to 10-week-old males or females; control mice were age- and sex-matched littermates. Mice were housed in either conventional or individually ventilated cages in temperature-controlled facilities on a 12 h:12 h light/dark cycle on standard diet. All mouse procedures were approved by the Imperial College London Ethical Committees and The Jackson Laboratory Institutional Animal Care and Use Committee and were in accordance with national and international regulations. The predominant mouse line used in this study was derived from a triple cross between C57BL/6J, FVB/NJ and NOD/ShiLtJ parental lines (CFN line). This line was obtained initially for a different study by mating Igf1r^fl/fl^ C57BL/6J mice (Jackson Laboratory, Bar Harbor, ME, USA) with Foxp3Cre transgenic NOD/ShiLtJ mice (Jackson Laboratory, Bar Harbor, ME, USA) Foxp3CreIgf1r^fl/fl^ mice were then crossed with IGF-1Ea transgenic FVB/NJ mice (Semenova et al., 2008). This triple cross was in long-term use for a different project (Johannesson et al., 2014) and was maintained by sibling mating for over 10 generations. Littermates not carrying the transgenes were used to establish the CFN line, which has been used for the present study. The line has been deposited at The Jackson Laboratories (JR29108).

### Resiquimod treatment

Mice were treated with TLR7 agonist Resiquimod (R848; Sigma-Aldrich, Dorset, UK) by topical application to the ear three times a week as previously described (Yokogawa et al., 2014). The treatment protocol was modified slightly to ensure dosing dependent on body mass (100 μg/30 μl per 30 g body mass which equals 3.4 μg/g body mass in 1:3 ethanol:acetone) and to adjust duration of treatment to the increased susceptibility of the CFN mice to treatment (2 weeks). Control mice were treated with 1 μl/g body mass 1:3 ethanol:acetone mix only. Tissue was generally harvested at week two, immediately after the end of the 2 week treatment protocol or at week four, after 2 weeks of recovery. Tissue was isolated after *in situ* perfusion with ice-cold phosphate-buffered saline (PBS, Sigma-Aldrich) through the apex of the left ventricle of the heart to clear blood from heart chambers and blood vessels.

### Macroscopic observations and scoring

After perfusion and excision of the heart, macroscopically visible haemorrhagic lesions were scored on a scale according to: 0, no visible lesions; 1, lesions cover <10% of heart surface; 2, lesions cover 10-30% of heart surface; 3, lesions cover 30–50% of heart surface; 4, lesions cover >50% of heart surface.

### Histology and scoring of damage parameters

Hearts of treated and untreated mice were excised after perfusion as described above and fixed in 4% formaldehyde overnight, dehydrated in an increasing gradient of ethanol and embedded in paraffin. Sections were cut at 5 µm and de-waxed and rehydrated in an ethanol gradient, then stained with Haematoxylin and Eosin (H&E) and PicoSirius Red/Fast Green. All reagents were purchased from Sigma-Aldrich. In order to allow efficient analysis of cardiac damage in a large number of samples, a system of semi-quantitative scores was established. H&E-stained sections were used to analyse and score eosinophilia of cardiomyocytes (as a sign of cardiomyocyte death because Eosin staining is stronger when cytoplasm gets more acidic), cardiomyocyte vacuolisation (sign of later stages of cardiomyocyte cell death), extravasation of red blood cells (sign of endothelial/capillary damage) and mononuclear cell infiltration (sign of the immune system causing and/or reacting to damage). PicoSirius Red staining was used to analyse and score fibrosis. Individual parameters were scored on a scale of 0 (none), 1 (mild), 2 (moderate) to 3 (severe) as shown in representative examples in Fig. S2. Scores were obtained from five fields of view along the myocardium at a 100× magnification on four midline cross-sections per animal by a blinded researcher. Images were captured using a LMD7000 microscope (Leica Microsystems, Milton Keynes, UK) and processed using the public domain software ImageJ (NIH; http://rsb.info.nih.gov) (Schneider et al., 2012).

### Echocardiography

Echocardiography was performed as described previously (Gallego-Colon et al., 2015; Stuckey et al., 2014) using a high-frequency ultrasound system Vevo 770 (VisualSonics, Toronto, Canada) with a 30 MHz linear transducer. Images were analysed using Vevo 770 workstation software. Mice were anaesthetised with 1-2% isofluorane to maintain heart rate at 450±50 bpm. Left ventricular ejection fraction (EF), fractional shortening (FS), mass (LV mass), end systolic volume (ESV) and end diastolic volume (EDV) were quantified using m-mode acquisitions in the parasternal short axis orientation. Diastolic function was assessed using 2D guided pulsed-wave Doppler acquisitions across the mitral valve in the apical four-chamber view.

### Magnetic resonance imaging

All MRI scans were performed at the Biological Imaging Centre, Imperial College, London. Longitudinal cardiac MRI was performed before, at week two and week four after start of treatment. Mice were anaesthetised with 1-2.5% isoflurane adjusted to maintain the respiratory rate of 65-75 breaths/min. Body temperature was maintained at 37±0.5°C by a heating mat. Respiration, ECG and body temperature were continuously monitored (SA Instruments, Stony Brook, NY, USA). MRI was performed on a 9.4 T-BioSpec system (Bruker BioSpin, Ettlingen, Germany) using an 86 mm inner diameter volume transmit quadrature coil and a mouse heart array receiver. EF, mass, ESV and EDV were quantified from a stack of ECG and respiratory-gated CINE gradient echo images in the short-axis plane (7 slices). The acquisition parameters for CINE measurements were: repetition time (TR)=RR interval/number of frames (∼10 ms for 11 frames), TR_effective_=RR interval, echo time (TE)=2.05 ms, flip angle=25°, slice thickness=1 mm (continuous slices), acquisition matrix=122×122, field of view=20×20 mm^2^, leading to a spatial in-plane resolution of 164×164 μm^2^. In order to ensure animal welfare during long anaesthetic periods for recovery MRI scans and to exclude passive functional effects on the heart, mice that did not show a severe clinical phenotype indicating systemic bleeding and decreased blood volume (ruffled fur, hunched posture, skin haemorrhages, decreased urinary output) were used for imaging.

### Disease transfer studies

Splenocytes and sera were isolated from CFN donor mice after 2 weeks of Resiquimod treatment and 1 week of recovery. Splenocytes were pre-stimulated *in vitro* with 5 mg/ml Concanavalin A (Sigma-Aldrich), washed thoroughly with PBS (Thermo Fisher Scientific, Loughborough, UK) and injected via the tail vein into healthy recipient CFN mice at 2×10^7^ cells/animal. Serum was stored immediately at −80°C and injected intraperitoneally at 200 μl/week for 3 weeks. Tissue was harvested 3 weeks after first injection and processed for histology as described above.

### Immune cell isolation from the heart and lymphoid tissues

For isolation of cells from heart, lymph nodes and spleen, mice were perfused *in situ* with ice-cold PBS through the apex of the left ventricle to clear blood from the circulation. To generate single-cell suspensions, tissues were excised, finely minced using surgical scissors on ice and digested with 0.25% collagenase F (Sigma-Aldrich) in RPMI medium for 30 min at 37°C with gentle agitation as described (Johannesson et al., 2014). After the first round of digestion, supernatants were filtered through 70 µm filters and transferred into 10% FBS and 300 U ml^−1^ DNaseI (Roche Diagnostics, Burgess Hill, UK) on ice to block enzyme activity and washed twice with ice-cold PBS for 5 min at 1200 rpm, 4°C. Undigested tissue pieces were transferred into fresh collagenase solution for a second round of digestion for 30 min at 37°C.

For *in vitro* culture experiments, excised spleens were gently mashed through a 70 μm cell strainer, washed and subjected to red blood cell lysis using Red Blood Cell Lysis Buffer (Sigma-Aldrich) according to the manufacturer's instructions. The obtained cell mixture was then used for flow cytometric analysis.

### *In vitro* Resiquimod stimulation

Isolated splenocytes were seeded at a concentration of 2×10^6^/ml in complete RPMI-1640 medium supplemented with 1% penicillin/streptomycin solution, 50 μM β-mercaptoethanol and 10% heat-inactivated foetal bovine serum (FBS); all purchased from Thermo Fisher Scientific. Cells were treated with 1 mg/ml Resiquimod daily for 4 days. Cells were visually inspected for signs of activation and harvested on day 4 for counting using a haemocytometer and flow cytometry analysis of cell populations.

### Flow cytometric analysis

Surface staining antibodies used were obtained from BioLegend (London, UK): anti-mouse CD45 [CD45-APC-Cy7, 103116, clone 30-F11, which reacts with all isoforms and both CD45.1 (NOD/ShiLtJ and FVB/NJ) and CD45.2 (C57Bl/6J) alloantigens, lot B185138, 1:800], anti-mouse CD3 (CD3-APC, 100235, clone 17A2, lot B166471, 1:200), anti-mouse CD19 (CD19-FITC, 115505, clone 6D5, lot B131781, 1:200), anti-mouse CD11b (CD11b-PE, 101207, clone M1/70, lot B166034, 1:400). Antibody dilutions in cell staining buffer containing 1% TruStain fcX (anti-mouse CD16/32) antibody were used to stain for pan surface markers of broad immune cell populations (T-cells, B-cells, myeloid cells). TLR7 expression was detected after permeabilisation of isolated splenocytes in 0.5% Triton X-100 in PBS, using a rabbit anti-mouse TLR7 antibody (Abcam, ab45371, 1:500) in MAXblock blocking solution (Active Motif, Carlsbad, CA, USA) and a goat anti-rabbit IgG-Alexa Fluor 488 secondary antibody (Thermo Fisher Scientific, A11008, 1:1000). Staining was performed following the manufacturer's instructions. Samples were acquired using a BD LSRII (Becton Dickinson, Oxford, UK) and analysed using Flow Jo v.9.8.5 (Treestar, Ashland, OR, USA) software (www.flowjo.com). Fluorescence minus one (FMO) controls were used for gating on individual populations as shown in Fig. S6.

### ELISA

ELISA assays were performed to detect anti-cardiac antibodies, to determine their isotypes and target antigens and to analyse the antibody isotype distribution in serum of Resiquimod-treated mice.

For anti-heart lysate, anti-myosin and anti-troponin assays, ELISA plates were coated overnight at 4^o^C with 1 μg/ml rat cardiac lysate (Novus Biologicals, Abington, UK), recombinant rat cardiac muscle myosin heavy chain MyHC-α, or recombinant rat cardiac troponin I (both from MyBioSource, San Diego, CA, US). Serum from Resiquimod-treated mice was used in serial dilutions (1:10, 1:100, 1:1000) in triplicate for relative quantification of antibodies against myosin and troponin. Secondary HRP-conjugated goat anti-mouse IgM (ab5930), IgG1 (ab97240), IgG2a (ab97245) or IgG2b (ab97250) all from Abcam were used at 1:10,000, and TMB Substrate (BioLegend) was used to detect binding.

For relative quantification of antibody isotypes in the serum, a Pierce Rapid Antibody Isotyping Kit - Mouse (Thermo Fisher Scientific) was used according to the manufacturer's instructions, using previously determined dilutions optimised for individual isotypes (Fig. S3). Optical densities were measured at 450 nm using a SpectraMAX190 microplate reader (Molecular Devices, Sunnyvale, CA, USA).

### Microscopy

For detection of antibodies and antibody/complement complex deposition in cardiac and kidney tissue *in vivo*, 5 µm sections of hearts and kidneys of Resiquimod-treated mice were stained with goat anti-mouse IgG-FITC (Sigma, F5387, lot 99H4872, 1:200), anti-mouse IgM-Alexa Fluor 488 (BioLegend, 406521, lot B203057, 1:200), or unlabelled anti-mouse IgG1/IgG2a/IgG2b/IgG3 (BioLegend, 406601/407101/406701/406802, 1:200) and a secondary goat anti-rat IgG-Alexa Fluor 488 (BioLegend, 405418, 1:500). C3 staining was performed using anti-mouse C3c-FITC (Thermo Fisher Scientific, PAI-29718, lot QL2122721H, 1:50), as described previously (Tarzi et al., 2002). Sections were counterstained with wheat germ agglutinin (WGA)-Alexa Fluor 594 (Thermo Fisher Scientific) to label membranes for 15 min at room temperature and mounted in Vectashield mounting liquid containing DAPI (Vector Laboratories, Peterborough, UK) to label nuclei.

Images were captured using a LMD7000 microscope (Leica microsystems) and processed using ImageJ.

### Statistics

Statistical planning of numbers was based on preliminary results of macroscopic haemorrhagic scoring (effect size 4). Aiming for a power of 0.95, group sizes of at least three were deemed sufficient to reach statistical significance. To account for potential experimental losses, at least 5 mice per group were used when possible. Power calculations were performed using G*Power 3.1 (Faul et al., 2007) available at http://www.gpower.hhu.de/. For MRI imaging, animals were selected as described in the MRI section. Scoring of histopathology was performed by a blinded researcher. Graph design and statistical analyses were performed using Prism (GraphPad Software). *P*-values are presented following GraphPad style as *P<0.05, **P<0.005, ***P<0.001 and ****P<0.0001. One- or two-tailed unpaired or paired Student's *t*-tests were performed as appropriate.
